# Laparoscopic Pectopexy Using a Cervical Cerclage Tape and Native Tissue Repair to Correct Vaginal Vault Prolapse

**DOI:** 10.1002/ccr3.70364

**Published:** 2025-04-01

**Authors:** Athanasios Protopapas, Nikolaos Kathopoulis, Athanasios Douligeris, Ioannis Chatzipapas, Themos Grigoriadis, Dimitrios Zacharakis, Konstantinos Kypriotis

**Affiliations:** ^1^ First Department of Obstetrics and Gynecology Alexandra' Hospital, National and Kapodistrian University of Athens Athens Greece

**Keywords:** laparoscopy, native tissue repair, pectopexy, pelvic organ prolapse

## Abstract

Laparoscopic pectopexy, facilitated by a cervical cerclage tape for the suspension of the vaginal vault, presents a feasible alternative to mesh sacrocolpopexy for the management of POP.

Pelvic organ prolapse (POP) represents a prevalent clinical condition with multiple clinical and psychological sequelae [1]. Anterior vaginal wall suspension to the pectineal ligament with mesh (pectopexy) is commonly performed when access to the promontory is challenging [2]. Video 1 illustrates the case of a 56‐year‐old woman with a post‐hysterectomy stage 3 apical prolapse alongside a stage 2 cystocele, managed with a new technique of laparoscopic pectopexy using a permanent cervical cerclage tape to suspend the vagina to the iliopectineal ligament. The tape was sutured to the vaginal wall over a reinforced anterior fascia after native tissue correction of the cystocele to reduce the attachment surface of the synthetic material to the vagina.

**VIDEO 1:** The video presenting an alternative technique for laparoscopic pectopexy without using a mesh, combined with laparoscopic native tissue correction of anterior and posterior defects. Video content can be viewed at https://onlinelibrary.wiley.com/doi/10.1002/ccr3.70364

## Question

Is there an alternative method to mesh sacrocolpopexy for the management of Pelvic Organ Prolapse?

## Author Contributions

**Athanasios Protopapas:** conceptualization, methodology. **Nikolaos Kathopoulis:** writing – original draft. **Athanasios Douligeris:** supervision, validation. **Ioannis Chatzipapas:** writing – review and editing. **Themos Grigoriadis:** methodology, supervision. **Dimitrios Zacharakis:** conceptualization, data curation. **Konstantinos Kypriotis:** conceptualization, resources.

## Ethics Statement

The method presented is exempt from institutional review board approval, as a common surgical step.

## Consent

Written informed consent was obtained from the patient to publish this report in accordance with the journal's patient consent policy.

## Conflicts of Interest

The authors declare no conflicts of interest.

## Data Availability

Data are available upon request.
